# CT Arthrography With Traction in Femoro-Acetabular Impingement: How to Do It?

**DOI:** 10.7759/cureus.67366

**Published:** 2024-08-21

**Authors:** Benjamin D Dallaudiere, Caroline Ziade, Pierre Abadie, Nicolas Bouguennec, Lionel Pesquer

**Affiliations:** 1 Radiology, Clinique du Sport de Bordeaux Mérignac, Bordeaux, FRA; 2 Orthopedic Surgery, Clinique du Sport de Bordeaux Mérignac, Bordeaux, FRA

**Keywords:** femoro-acetabular impingement, traction, ct scan, arthrography, hips

## Abstract

Computed tomography arthrography (CTA) highly correlates with arthroscopy in detecting labral tears, especially in patients with positive impingement tests. CTA enables the acquisition of isotropic datasets with high spatial resolution within a single acquisition. However, the hip is a close-fitting, congruent, and nearly spherical joint, with relatively thin cartilage. We describe herein a simple and cost-effective method using traction for hip CTA, with axial manual distraction, that helps overcome the usual limitations, by widening the articular joint space and thus better delineating both acetabular and femoral cartilages.

## Introduction

Computed tomography arthrography (CTA) has already proved its efficiency in detecting labral tears with arthroscopic findings correlation (sensitivity and specificity around 90%), in patients with positive impingement tests [1]. CTA has an advantage in that it enables the acquisition of isotropic datasets with high spatial resolution within a single acquisition. The hip joint is characterized by its close fit, congruence, and nearly spherical shape, featuring relatively thin cartilage. Employing CTA alongside traction offers a straightforward and economically viable approach to expanding the intra-articular joint space, thereby facilitating the visualization of both acetabular and femoral cartilage [2]. The purpose of this article is first to describe CTA with traction technique and second to briefly describe the grading of chondral and labral lesions of this easy procedure in patients with clinical positive impingement correlated with arthroscopic data gold standard.

## Technical report

The injection was administered anterolaterally into the joint, ensuring sterility, and guided by fluoroscopy employing a 21 G needle. The injected volume contained 1 mL of local anesthetic (lidocaine hydrochloride, Xylocaine®, 10 mg/mL, AstraZeneca, London, United Kingdom) and 11 mL of an iodinated contrast agent (iodixanol, Visipaque®, 270 mg/mL, GE Healthcare, Chicago, or ioxaglate meglumine, Hexabrix®, 320 mg/mL, Guerbet, Villepinte, France). It is crucial to expel any trapped air in the needle before proceeding, as it could potentially interfere with interpretation. Additionally, an excessive volume of contrast injected may lead to diffusion in soft tissues, potentially impacting the analysis of extracapsular structures.

Then, the subject was encouraged to walk and mobilize the hip before CTA for about five minutes. Traction was applied, which was made by putting the ankle in a specially manufactured boot to which weights of 16-34 kg were attached, depending on the patient's weight (16, 28, and 34 kg for patients under 55, between 55 and 65, and others, respectively) and the supported traction. The leg was extended caudally with the hip in neutral rotation.

CT images were acquired five minutes after traction beginning with a 16 slices machine (Optima CT 580w, General Electric Healthcare, Chicago). The scan area was adjusted with a scout image to a field of view centered on the hip. The acquisition parameters were a rotation speed of 5.62 mm/rotation, a current of 350 mAs, a voltage of 140 kV, a collimation of 10 mm, and a slice thickness of 0.625 mm.

Before applying weight, it is crucial to conduct manual axial distraction of the hip joint, enabling continuous traction and ensuring consistent distraction of articular spaces.

**Figure 1:**
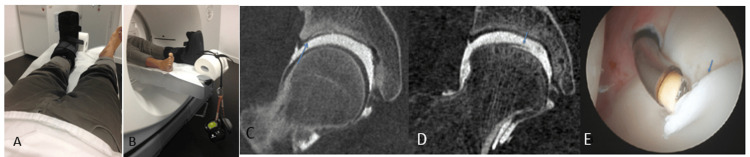
Hip positioning (A and B) and representative chondral (C-E) Traction was applied on a house-manufactured boot with 34 kg weights. The leg is extended caudally with the hip in neutral rotation (A and B). Coronal reformation of CTA of the right hip with traction in different patients with acetabular chondral flap (arrow) (C) and femoral chondral flap (arrow) (D). E (arrow) represents the arthroscopic correlation for the findings in D. CTA: computed tomography arthrography

Without prior manual distraction, axial traction proves ineffective. Furthermore, patient education regarding the procedure is vital to promote relaxation and enhance cooperation (Figure 1 and Video 1).

**Video 1:** CTA procedure CTA: computed tomography arthrography

## Discussion

Based on our observations, axial traction enhances the visibility of articular spaces and improves contrast material coverage of cartilage, consistent with previous findings. However, there remains a lack of consensus regarding the optimal amount of traction required, with reported ranges varying from 6 to 25 kg. It depends on the patient's weight, the traction force supported, and above all the morphology of the hip [2-5]. Indeed, normal hips or patients with acetabular retroversion and impingement likely require greater traction force than patients with hip dysplasia. This traction is well accepted by patients but sometimes causes pain during and after the procedure [6].

The performance of CTA without traction was studied, with a very good specificity between 90% and 100% for cartilage or labrum involvement [1]. So, it detects the undamaged hip well. However, its sensitivity is limited; it drops to 88% for acetabular cartilage, with its corollary of false negatives [3,7]. Radiologists then became interested in traction to allow decoaptation and improve the diagnostic performance of imaging. Indeed, without traction, cartilage images are often the sum of femoral and acetabular coatings, and decoaptation by traction to better typify and unmask cartilage lesions.

Traction equipment used in the literature varies from one team to another. However, several have used similar systems in our study, although often the weights put in place are lower [8-10]. In the study by Henak et al. [2], who proposed different traction according to the various hip pathologies of their cohort, the average joint distraction after traction was 2.46 versus 4.2 mm in our series. Some authors have studied pain during examination. More than half of the patients described pain during the injection or the traction, which disappeared after 24 hours [5,6].

**Figure 2:**
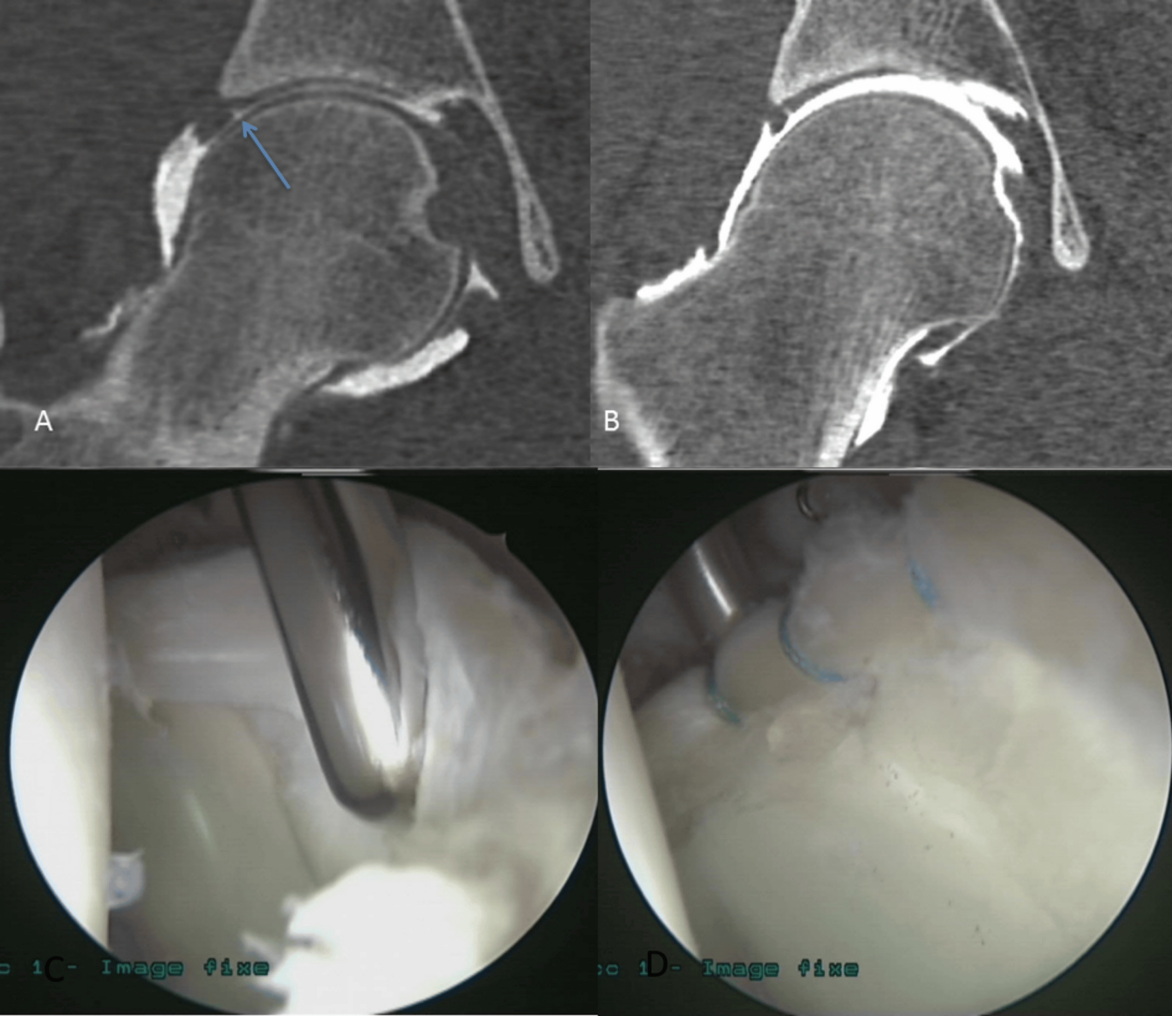
Labral lesions CTA of the right hip with labral-chondral separation without (A) and with (B) traction in the same patient 1 (MAHORN grade 1). These coronal reformations of CTA of the right hip show better labral-chondral separation assessment (arrow) with traction than without traction in the same patient. Arthroscopy comparison before (C) and after (D) suture treatment in patient 2. CTA: computed tomography arthrography, MAHORN: Multicenter Arthroscopy of the Hip Outcomes Research Network

In our opinion, CTA with traction also makes a better assessment for chondral and labral degenerative, traumatic, or micro-traumatic lesions. Maximizing axial traction is advisable during hip CTA for femoro-acetabular impingement, as it elevates the examination's quality and aids in identifying labral and chondral tears. Additionally, it proves simple to execute and is generally well tolerated by patients (Figure 2).

Indeed, hip imaging has a delicate interpretation because of the fineness of the joint space and the small size of the initial labral and cartilage lesions. The challenge of imaging in the femoro-acetabular impingement is to detect the lesions as soon as possible to better adapt the care of the patient, especially the athlete.

In our clinical practice, the modified Multicenter Arthroscopy of the Hip Outcomes Research Network (MAHORN) classification allowing to determine a grade of labrum and cartilage lesions was used in both imaging and surgery [6]. When multiple lesions were evident within the joint, the most severe damage was recorded and utilized for grading purposes. Detection of cartilage delamination was required on two planes for assessment.

## Conclusions

In conclusion, CTA with traction is an easy and efficient technique concerning the detection and grading of chondral and labral lesions, according to surgery data for the hip impingement diagnosis.
